# Supplementary material for: Characterizing Disparities in the HIV Care Continuum among Transgender and Cisgender Medicare Beneficiaries
Source: medRxiv. 2024 Mar 20:2024.03.19.24304525. Preprint. [Version 1] doi: 10.1101/2024.03.19.24304525 (PMC10984057; doi:10.1101/2024.03.19.24304525)
Supplement: 1 [file NIHPP2024.03.19.24304525V1-supplement-1.pdf]

Supplement Table 1. Unadjusted prevalence of HIV Care Engagement Outcomes among transgender and gender diverse (TGD) (n=1,678) and cisgender (n=10,681) Medicare beneficiaries with HIV, overall and by gender subgroup, 2008-2017.

	TGD						Cisgender				Total			
	TFN		TMN		Unclassified		Male		Female		TGD		Cisgender	
**HIV Care Visits**	**N=625**	**%**	**N=78**	**%**	**N=232**	**%**	**N=3,719**	**%**	**N=1,747**	**%**	**N=935**	**%**	**N=5,466**	**%**
2008	97	19.4%	12	19.4%	18	13.3%	524	16.9%	273	20.2%	127	18.2%	797	17.9%
2009	120	21.1%	15	18.5%	37	24.0%	578	16.9%	297	18.7%	172	21.4%	875	17.5%
2010	146	23.1%	11	12.8%	33	17.7%	668	17.7%	350	19.3%	190	21.0%	1018	18.2%
2011	153	21.8%	19	18.1%	47	21.8%	782	19.1%	382	19.0%	219	21.4%	1164	19.0%
2012	176	23.1%	17	14.2%	43	17.7%	848	19.2%	390	18.0%	236	21.0%	1238	18.8%
2013	213	26.7%	22	16.4%	67	24.0%	1035	22.1%	459	19.6%	302	24.9%	1494	21.3%
2014	220	28.2%	17	13.2%	64	21.8%	1111	23.4%	472	19.8%	301	25.0%	1583	22.2%
2015	216	28.0%	25	19.5%	77	25.6%	1223	25.0%	537	21.7%	318	26.5%	1760	23.9%
2016	242	30.7%	31	24.4%	72	22.2%	1399	27.5%	607	23.9%	345	27.8%	2006	26.3%
2017	222	29.6%	26	20.8%	87	26.0%	1386	26.6%	581	23.1%	335	27.7%	1967	25.4%
**STI Screening**	**N=541**	**%**	**N=62**	**%**	**N=168**	**%**	**N=2,028**	**%**	**N=951**	**%**	**N=771**	**%**	**N=2,979**	**%**
2008	63	12.6%	4	6.5%	14	10.4%	192	6.2%	88	6.5%	81	11.6%	280	6.3%
2009	87	15.3%	8	9.9%	15	9.7%	265	7.8%	128	8.1%	110	13.7%	393	7.9%
2010	96	15.2%	9	10.5%	16	8.6%	343	9.1%	149	8.2%	121	13.4%	492	8.8%
2011	134	19.1%	14	13.3%	25	11.6%	400	9.8%	190	9.4%	173	16.9%	590	9.7%
2012	173	22.7%	16	13.3%	35	14.4%	502	11.4%	177	8.2%	224	19.9%	679	10.3%
2013	196	24.5%	19	14.2%	41	14.7%	593	12.7%	220	9.4%	256	21.1%	813	11.6%
2014	187	24.0%	17	13.2%	38	12.9%	617	13.0%	241	10.1%	242	20.1%	858	12.0%
2015	202	26.2%	25	19.5%	52	17.3%	644	13.2%	259	10.5%	279	23.3%	903	12.3%
2016	203	25.7%	26	20.5%	71	21.9%	685	13.5%	247	9.7%	300	24.2%	932	12.2%
2017	194	25.9%	26	20.8%	72	21.5%	751	14.4%	274	10.9%	292	24.1%	1025	13.2%
**Prescribed ART**	**N=879**	**%**	**N=89**	**%**	**N=365**	**%**	**N=4,838**	**%**	**N=1,759**	**%**	**N=1,333**	**%**	**N=6,597**	**%**
2008	306	64.3%	22	37.9%	88	70.4%	1687	54.6%	514	38.0%	416	63.1%	2201	49.5%
2009	372	68.8%	27	35.5%	100	70.9%	1898	55.6%	588	37.1%	499	65.8%	2486	49.7%
2010	439	73.3%	27	34.6%	115	69.3%	2098	55.5%	683	37.7%	581	68.9%	2781	49.7%
2011	484	72.8%	39	40.2%	139	70.2%	2262	55.2%	739	36.7%	662	69.0%	3001	49.1%
2012	561	77.5%	44	39.3%	161	72.9%	2596	58.8%	867	40.0%	766	72.5%	3463	52.6%
2013	592	77.2%	49	39.2%	184	71.0%	2793	59.7%	944	40.4%	825	71.7%	3737	53.2%
2014	572	76.5%	52	43.0%	200	73.5%	2853	60.0%	991	41.6%	824	72.2%	3844	53.9%
2015	560	75.8%	54	45.0%	209	75.7%	2945	60.3%	1081	43.7%	823	72.5%	4026	54.7%
2016	591	77.9%	53	44.5%	224	75.9%	3213	63.1%	1149	45.2%	868	74.0%	4362	57.1%

2017	576	79.8%	62	53.4%	237	78.0%	3392	65.0%	1205	47.8%	875	76.6%	4597	59.4%
**ART Persistence: 90%**	**N=576**	**%**	**N=47**	**%**	**N=208**	**%**	**N=3,237**	**%**	**N=1,059**	**%**	**N=831**	**%**	**N=4,296**	**%**
2008	94	19.7%	5	8.6%	31	24.8%	619	24.0%	172	15.2%	130	19.7%	791	21.3%
2009	134	24.8%	10	13.2%	32	22.7%	814	28.6%	225	16.7%	176	23.2%	1,039	24.8%
2010	163	27.2%	12	15.4%	47	28.3%	917	28.9%	267	17.0%	222	26.3%	1,184	25.0%
2011	201	30.2%	9	9.3%	39	19.7%	977	28.0%	292	16.5%	249	25.9%	1,269	24.2%
2012	239	33.0%	14	12.5%	55	24.9%	1,155	30.3%	345	17.8%	308	29.1%	1,500	26.1%
2013	253	33.0%	22	17.6%	71	27.4%	1,288	31.5%	408	19.0%	346	30.1%	1,696	27.2%
2014	256	34.2%	20	16.5%	79	29.0%	1,341	32.3%	404	18.5%	355	31.1%	1,745	27.6%
2015	251	34.0%	25	20.8%	89	32.2%	1,384	32.9%	466	20.6%	365	32.2%	1,850	28.6%
2016	268	35.3%	21	17.6%	80	27.1%	1,524	34.4%	519	22.2%	369	31.5%	2,043	30.2%
2017	284	39.3%	28	24.1%	103	33.9%	1,663	36.9%	545	23.6%	415	36.3%	2,208	32.4%
**ART Persistence: 95%**	**N=429**	**%**	**N=31**	**%**	**N=147**	**%**	**N=2,342**	**%**	**N=726**	**%**	**N=607**	**%**	**N=3,068**	**%**
2008	48	10.1%	3	5.2%	14	11.2%	301	11.7%	82	7.3%	65	9.9%	383	10.3%
2009	74	13.7%	4	5.3%	17	12.1%	383	13.5%	111	8.2%	95	12.5%	494	11.8%
2010	84	14.0%	6	7.7%	24	14.5%	437	13.8%	140	8.9%	114	13.5%	577	12.2%
2011	99	14.9%	3	3.1%	19	9.6%	493	14.2%	137	7.7%	121	12.6%	630	12.0%
2012	124	17.1%	7	6.3%	21	9.5%	599	15.7%	171	8.8%	152	14.4%	770	13.4%
2013	120	15.6%	8	6.4%	37	14.3%	605	14.8%	183	8.5%	165	14.3%	788	12.7%
2014	117	15.6%	12	9.9%	42	15.4%	649	15.6%	197	9.0%	171	15.0%	846	13.4%
2015	132	17.9%	14	11.7%	36	13.0%	658	15.6%	235	10.4%	182	16.0%	893	13.8%
2016	143	18.8%	14	11.8%	43	14.6%	796	18.0%	270	11.6%	200	17.1%	1,066	15.8%
2017	142	19.7%	10	8.6%	48	15.8%	818	18.1%	242	10.5%	200	17.5%	1,060	15.6%

Note. The percentages reflect the row total for a given year. TGD= Transgender and gender diverse; TFN= trans feminine and non-binary; TMN = trans masculine and non-binary; Unclassified = gender category could not be determined. ART = Antiretroviral. STI= Sexually Transmitted Infections.

medRxiv preprint doi: https://doi.org/10.1101/2024.03.19.24304525; this version posted March 20, 2024. The copyright holder for this preprint (which was not certified by peer review) is the author/funder, who has granted medRxiv a license to display the preprint in perpetuity.
It is made available under a CC-BY 4.0 International license .

Supplement Table 2. Predicted probabilities of HIV Care Continuum engagement among transgender and gender diverse (TGD) (n=1,678) and cisgender (n=10,681) Medicare beneficiaries with HIV, overall and by gender subgroup, 2008-2017.

Gender	Estimate	SE	Lower	Upper
**HIV Care Visits**				
TFN	0.25	0.009	0.24	0.27
TMN	0.18	0.020	0.14	0.22
Unclassified	0.22	0.014	0.20	0.25
TGD	0.23	0.007	0.22	0.24
Cisgender	0.22	0.003	0.21	0.22
Cisgender Male	0.22	0.003	0.22	0.23
Cisgender Female	0.21	0.005	0.20	0.22
**STI Screening**				
TFN	0.22	0.009	0.21	0.24
TMN	0.14	0.019	0.10	0.18
Unclassified	0.15	0.012	0.13	0.17
TGD	0.12	0.003	0.11	0.12
Cisgender	0.09	0.003	0.09	0.10
Cisgender Male	0.16	0.006	0.15	0.17
Cisgender Female	0.11	0.002	0.11	0.12
**Prescribed ART**				
TFN	0.71	0.012	0.69	0.74
TMN	0.41	0.034	0.34	0.47
Unclassified	0.68	0.020	0.64	0.71
TGD	0.61	0.011	0.59	0.63
Cisgender	0.52	0.004	0.52	0.54
Cisgender Male	0.58	0.005	0.57	0.59
Cisgender Female	0.40	0.008	0.39	0.42
**ART: 90% Persistence**				
TFN	0.30	0.010	0.28	0.32
TMN	0.15	0.023	0.11	0.20
Unclassified	0.25	0.016	0.22	0.28
TGD	0.27	0.008	0.25	0.28
Cisgender	0.23	0.003	0.22	0.23
Cisgender Male	0.26	0.004	0.25	0.27
Cisgender Female	0.17	0.005	0.16	0.18
**ART: 95% Persistence**				
TFN	0.15	0.007	0.14	0.16
TMN	0.07	0.014	0.04	0.10
Unclassified	0.12	0.010	0.10	0.14
TGD	0.13	0.005	0.12	0.14
Cisgender	0.11	0.002	0.11	0.12
Cisgender Male	0.13	0.003	0.12	0.13
Cisgender Female	0.08	0.003	0.07	0.09

Note. TGD = Transgender and gender diverse; TFN = trans feminine and non-binary; TMN = trans masculine and non-binary; Unclassified = gender category could not be determined. SE= Standard Error. Lower and Upper = Lower and Upper 95% Confidence Intervals. STI = Sexually Transmitted Infection. ART = Antiretroviral medication.

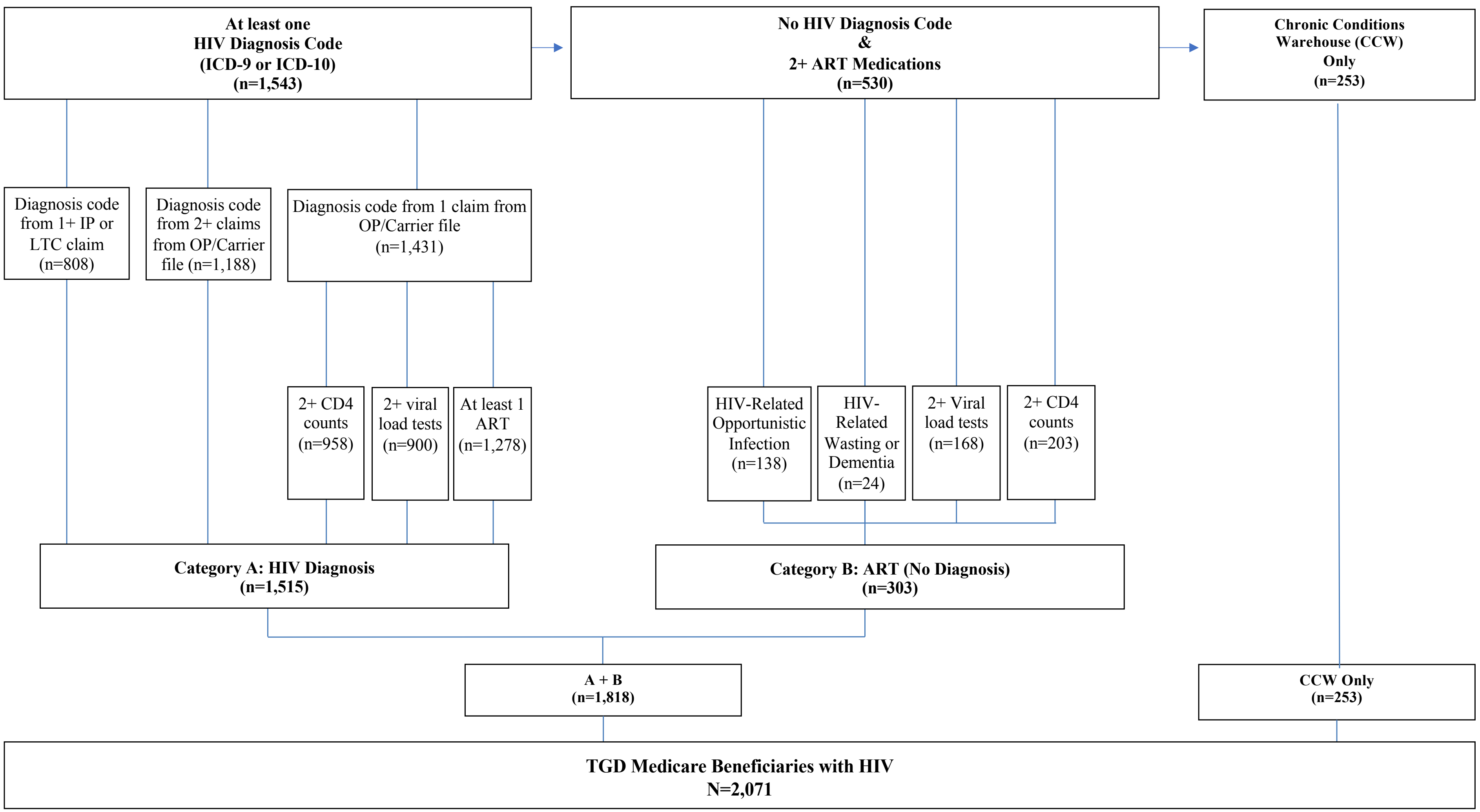

Supplement Fig 1. Transgender and Gender Diverse (TGD) Medicare Beneficiaries with HIV, 2008-2017. Flow diagram of TGD sample. The following individuals were excluded: individuals under 18 and those who died or did not have continuous Fee-for-Service coverage for at least 6 months of the performance year, leaving a final analytic sample of N=10,681. IP=inpatient; OP=outpatient; LTC=long term care; ART=antiretroviral medication; ICD=International Classification of Diseases.

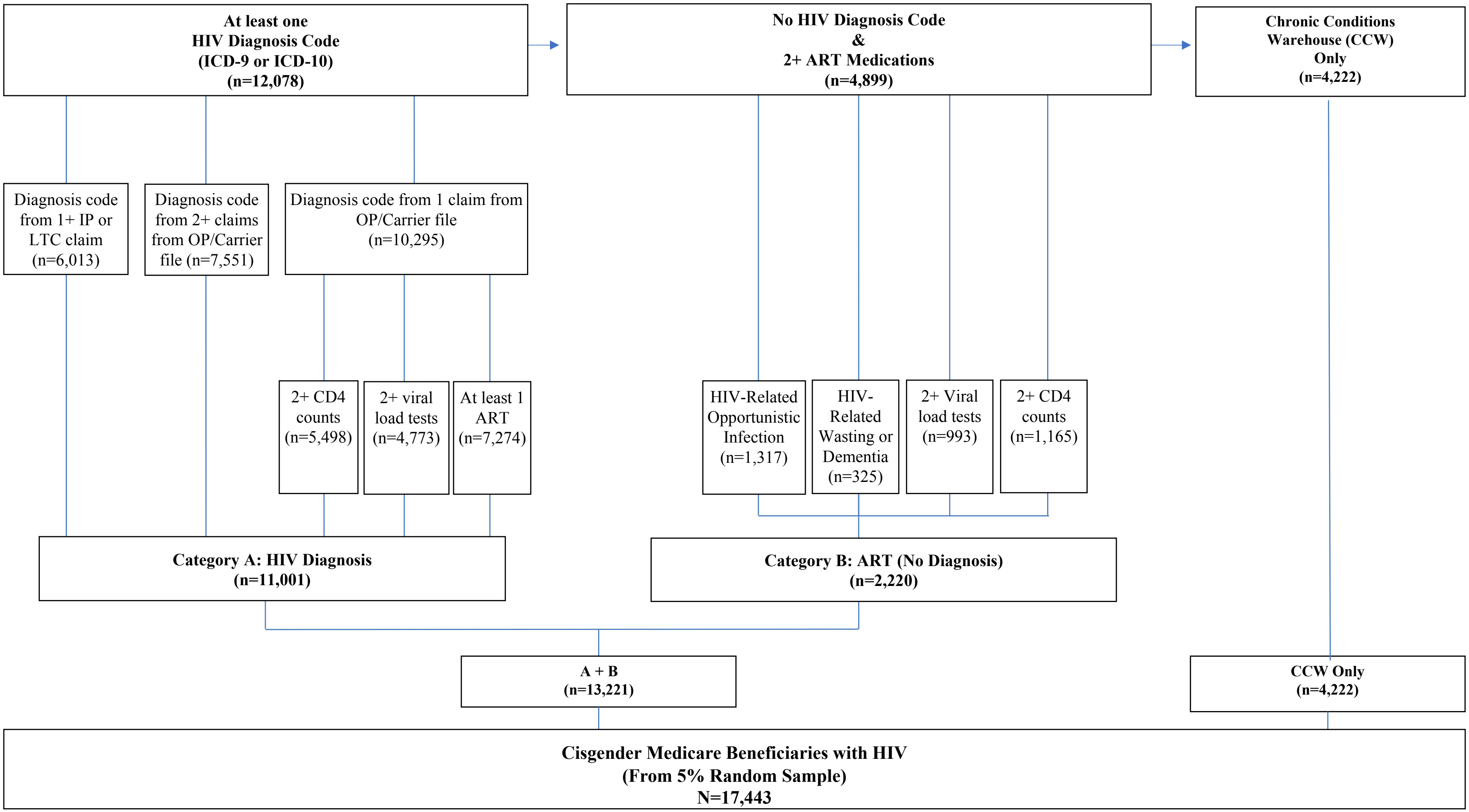

Supplement Figure 2: Cisgender Medicare Beneficiaries with HIV, 2008-2017. Flow diagram of TGD sample. The following individuals were excluded: individuals under 18 and those who died or did not have continuous Fee-for-Service coverage for at least 6 months of the performance year, leaving a final analytic sample of N=10,681. IP=inpatient; OP=outpatient; LTC=long term care; ART=antiretroviral medication; ICD=International Classification of Diseases.
